# A decade of genomic and phenotypic adaptation of carbapenem-resistant *Acinetobacter baumannii*

**DOI:** 10.3389/fcimb.2025.1527488

**Published:** 2025-04-30

**Authors:** Astri D. Tagueha, Cartesio D’Agostini, Daniela Scribano, Carlotta Fiorilla, Dolores Limongi, Silvia Fillo, Luca Corrent, Martina Lipari, Florigio Lista, Lucia Nencioni, Anna Teresa Palamara, Cecilia Ambrosi

**Affiliations:** ^1^ Department of Public Health and Infectious Diseases, Sapienza University of Rome, Rome, Italy; ^2^ Department of Experimental Medicine, University of Rome Tor Vergata, Rome, Italy; ^3^ Laboratory of Clinical Microbiology, Policlinico Tor Vergata, Rome, Italy; ^4^ Department of Promotion of Human Sciences and Quality of Life, San Raffaele Open University, Rome, Italy; ^5^ Laboratory of Microbiology, Istituto di Ricovero e Cura a Carattere Scientifico (IRCCS) San Raffaele Roma, Rome, Italy; ^6^ Scientific Department, Army Medical Center, Defense Institute for Biomedical Sciences, Rome, Italy; ^7^ Department of Public Health and Infectious Diseases, Laboratory Affiliated to Institute Pasteur Italia-Cenci Bolognetti Foundation, Sapienza University of Rome, Rome, Italy; ^8^ Department of Infectious Diseases, Istituto Superiore di Sanità, Rome, Italy

**Keywords:** *Acinetobacter baumannii*, motility, biofilm, desiccation, host-pathogen interaction, phenotypic and genotypic comparison

## Abstract

**Introduction:**

*Acinetobacter baumannii* exhibits high genomic plasticity, enabling it to acquire virulence factors and antibiotic resistance (AR). Understanding its evolutionary adaptations is crucial for developing effective therapeutic strategies.

**Methods:**

Thirty clinical isolates collected from two distinct time periods, defined as older (2010–2013), and recent (2022–2023),- were compared phenotypically (antibiotic resistance, growth, biofilm formation, desiccation tolerance, invasiveness) and genotypically (whole-genome sequencing).

**Results:**

All isolates displayed an extensively drug-resistant phenotype. Overall, respiratory isolates harbored a higher content of antibiotic-resistant genes (ARGs), with older isolates showing 12.5% increases in the average number of ARGs compared to recent urine isolates (*P* = 0.02). More than 50% of the strains with faster growth, stronger biofilm formation, and increased lung cell invasiveness were recent respiratory isolates, while over 70% of older isolates showed greater desiccation tolerance and bladder cell invasiveness. Eleven virulence factor genes were shared between old and recent respiratory isolates, and eight were common between recent urinary and respiratory strains with no overlap among urinary isolates. Statistically significant positive correlations were observed between fast-growing and strong biofilm-forming respiratory isolates as well as their lung cell invasiveness. Conversely, negative correlations were found between collection time, isolation site, and host cell invasiveness. Analysis of macrocolony types revealed no link to phenotypic behavior.

**Conclusion:**

Significant genetic variability was found between past and recent isolates. Older isolates had more genes involved in adhesion and nutrient uptake, while recent respiratory strains demonstrated increased biofilm formation and invasiveness, reflecting adaptation to clinical pressures. These findings highlight the dynamic evolution of *A. baumannii*, providing insights for future therapeutic strategies and infection control.

## Introduction

*Acinetobacter baumannii* is a major opportunistic pathogen responsible for severe nosocomial infections worldwide, primarily affecting the lungs and bloodstream; however, the number of isolates collected from urinary sites has recently increased significantly compared to the past (Duan et al., 2024; Obenhuber et al., 2024), raising the possibility that either it was previously underestimated or that *A. baumannii* isolates adapted to urinary tract. Currently, respiratory and urinary isolates exhibit robust environmental and host persistence and several antibiotic resistance (AR) mechanisms (Yang et al., 2019; Zeighami et al., 2019). Moreover, transmission is not limited to vulnerable and critically ill patients in intensive care units (ICUs), but also to patients in general wards with long-term care treatment (Valencia-Martín et al., 2019; Alrahmany et al., 2022). According to European Antimicrobial Resistance Surveillance Network (EARS-Net) in 2023, 59% of the 8,429 *Acinetobacter* spp. invasive isolates collected from 25 European countries exhibited resistance to multiple classes of antibiotics (www.ecdc.europa.eu/en). As global mortality associated with multidrug-resistant (MDR) *A. baumannii* accounted for 132.000 cases in 2019 (www.iris.who.int) and is projected to increase further, new medical therapeutic countermeasures for this pathogen have been prioritized (Murray et al., 2022; Hu et al., 2023).

In the last decades, *A. baumannii* clinical isolates have been exposed to enormous stress conditions, particularly due to the extensive use of antibiotics. As a consequence, *A. baumannii* isolates undergo local clonal expansion and adapt progressively to environmental and host changes (Ambrosi et al., 2017; Ambrosi et al., 2020; Stracquadanio et al., 2022; Scribano et al., 2024). *A. baumannii* great genomic plasticity allowed the acquisition of a remarkable number of AR genes (ARGs) via horizontal gene transfer through plasmid splicing, integrons, transposons, and mutations (Hamidian and Hall, 2021; Ambrose et al., 2023). Other mechanisms, such as changes in the outer membrane proteins, target modification, and efflux pumps, further promote *A. baumannii* extraordinary resistance to antibiotics (Sarshar et al., 2021; Cain and Hamidian, 2023; Scribano et al., 2024). Acquirable traits allow *A. baumannii* isolates to adapt and persist in healthcare settings by forming biofilms, tolerating desiccation, improving motility and invasiveness. Isolates that are more adapted to local stresses expand clonally; currently, eleven international clones (IC) have been identified (Hansen et al., 2023; Karah et al., 2023). From epidemiological studies, it is evident that IC2 is the predominant and more spread clone, especially in Europe, whereas the others show a particular geographic dominance (Hansen et al., 2023; Karah et al., 2023; Müller et al., 2023). Several evidence demonstrated the fast evolution of this opportunistic pathogen in the healthcare environments and in the host during infection driven by stress exposure to achieve maximal adaptation (Holt et al., 2019; Penesyan et al., 2019; Pompilio et al., 2021). Therefore, the present study aimed to genotypically and phenotypically characterize a collection of endemic carbapenem-resistant *A. baumannii* clinical isolates, collected in two distinct periods nearly a decade apart to identify key features, including antibiotic resistance and virulence traits, to understand their successful microevolution in the nosocomial environment.

## Methods

### Bacterial isolates, growth curves, and antibiogram profiles

Thirty *A. baumannii* carbapenem-resistant isolates were collected from two periods (2010–2013) and (2022– 2023) from three hospitals in Rome, Italy, including Policlinico Umberto I (n=9), Policlinico Di Liegro (n=6), and Policlinico Tor Vergata (n=15). Inclusion criteria: Carbapenem-resistant *A. baumannii* strains isolated from respiratory or urinary specimens, reflecting prevalent infection sources and the high risk of progression to pan-drug resistance. Exclusion criteria: Strains not exhibiting carbapenem resistance; strains isolated from other clinical sources. A single *A. baumannii* strain per patient was included in this study and was isolated mainly from respiratory specimens, including sputum (n=6), bronchoalveolar lavage (BAL) (n=4), bronchoalveolar aspirate (BA) (n=12), whereas 8 from urinary infections (**Table 1**). Species were identified by MALDI-TOF mass spectrometry. Strains were grown on Luria-Bertani (LB) broth and agar plates (LA). Growth kinetics in 96 well microplates were determined by measuring cell density (OD_600_) for 16 h at 37°C in LB with vigorous shaking (200 rpm) in triplicate wells for each strain, using the plate reader BioTek SynergyHT equipped with the software GEN5™. Three independent experiments were performed. The reference strains ATCC 17978 and ATCC 19606 were used as controls for all the assays, while strain AB5075 for surface-associated motility only (Scribano et al., 2019). Antibiogram profiles were obtained using the VITEK^®^2 system (bioMérieux, Italia S.p.A, Grassina, Italy) with the AST-N397 panel, which included amikacin, gentamicin, tobramycin, imipenem, meropenem, piperacillin/tazobactam, ciprofloxacin, trimethoprim/sulfamethoxazole, and colistin. Minimum inhibitory concentration (MIC) values were interpreted according to the European Committee on Antimicrobial Susceptibility Testing (EUCAST v13.0, 2023). Detailed MIC values and breakpoints are provided in **Table 2**.”

**Table 1:** Strains used in the study.

Site of isolation	Strain ID	Year	Strain ID	Year
Sputum	SN11	2022	SO3	2012
	SN12	2022	SO5	2012
	SN24	2023	SO28	2010
BL	BLN17	2023	BLO34	2012
	BLN19	2023	BLO70	2010
BA	BN14	2022	BO27	2012
	BN15	2022	BO29	2010
	BN20	2023	BO47	2011
	BN21	2023	BO88	2010
	BN22	2023	BO90	2010
	BN25	2023	BO93	2010
Urine	UN16	2022	UO2	2012
	UN18	2023	UO4	2013
	UN23	2023	UO7	2013
	UN26	2023	UO8	2013

BL, bronchoalveolar lavage; B, bronchial aspirate; U, urine; O, old collection; N, new collection.

**Table 2:** MIC values of clinical isolates.

	Aminoglycosides			Carbapenems		β-lactams	Quinolones	Sulfonamides	Polymyxins
Strain ID	GM	TM	AK	IMP	MRP	P/T	CIP	T/S	CST*
SO3	≥16	≥16	≤2*	≥16	≥16	≥128	≥4	≥320	≤0.5
SO5	≥16	≥16	32	≥16	≥16	≥128	≥4	≥320	2
SO28	≥16	≥16	≥64	≥16	≥16	≥128	≥4	≥320	≤0.5
SN11	≥16	≥16	≥64	≥16	≥16	≥128	≥4	≥320	≤0.5
SN12	8	≤1*	4*	≥16	≥16	≥128	≥4	≥320	≤0.5
SN24	≥16	≥16	≥64	≥16	≥16	≥128	≥4	≥320	≤0.5
BO27	≥16	≥16	≥64	≥16	≥16	≥128	≥4	≥320	≤0.5
BO29	≥16	≥16	≥64	≥16	≥16	≥128	≥4	≥320	≤0.5
BO47	≥16	≥16	≥64	≥16	≥16	≥128	≥4	≥320	≤0.5
BO88	≥16	≥16	≥64	≥16	≥16	≥128	≥4	160	≤0.5
BO90	≥16	≥16	≥64	≥16	≥16	≥128	≥4	≥320	≤0.5
BO93	8	≥16	8*	≥16	≥16	≥128	≥4	≥320	≤0.5
BN14	≥16	≥16	≤2*	≥16	≥16	≥128	≥4	≥320	≤0.5
BN15	>8	≥16	≤4*	≥16	≥16	≥128	≥4	≥320	≤0.5
BN20	≥16	≥16	≥64	≥16	≥16	≥128	≥4	≥320	≤0.5
BN21	≥16	≥16	8*	≥16	≥16	≥128	≥4	≥320	1
BN22	≥16	≥16	≥64	≥16	≥16	≥128	≥4	≥320	≤0.5
BN25	≥16	≥16	≥64	≥16	≥16	≥128	≥4	≥320	≤0.5
BLO34	≥16	≥16	≥64	≥16	≥16	≥128	≥4	≥320	≤0.5
BLO70	≥16	≥16	32	≥16	≥16	≥128	≥4	≥320	≤0.5
BLN17	≥16	≥16	≥64	≥16	≥16	≥128	≥4	160	≤0.5
BLN19	>8	≥16	>16	≥16	16	≥128	>1	≥320	≤0.5
UO2	≥16	≥16	≥64	≥16	≥16	≥128	≥4	≥320	≤0.5
UO4	≥16	≥16	≥64	≥16	≥16	≥128	≥4	≤20*	1
UO7	≥16	≥16	≥32	≥16	≥16	≥128	≥4	≥320	2
UO8	≥16	≥16	≥64	≥16	≥16	≥128	≥4	≥320	≤0.5
UN16	≥16	≥16	≥32	≥16	≥16	≥128	≥4	≥320	≤0.5
UN18	≥16	≥16	≥64	≥16	≥16	≥128	≥4	≥320	≤0.5
UN23	≥16	≥16	≥64	≥16	≥16	≥128	≥4	≥320	≤0.5
UN26	≥16	≥16	4*	≥16	≥16	≥128	≥4	≥320	≤0.5

AK, Amikacin (R>8); GM, Gentamicin (R>4); TM, Tobramicin (R>4); IMP, Imipenem R(>2); MRP, Meropenem (R>8); P/T, Piperacilin-tazobactam; CIP, Ciprofloxacin (R>1); T/S, Trimethoprim/Sulfamethoxazole (R>80); CST, Colistin (R>2). Asterix (*) indicated the susceptibility to the tested antibiotic. The classification was based on EUCAST clinical breakpoints (v13.0).

### Whole genome sequencing, molecular typing, antibiotic and virulence factors profiles

DNA was extracted using a Bacterial Genomic Isolation Kit (BioVision, USA). Libraries were prepared using the Nextera XT DNA kit and were sequenced on Illumina NextSeq500 and NextSeq2000 platforms using High Output Kit v2.5 (300 cycles) and P2 reagents (300 cycles), respectively; following the manufacturer’s instructions (Illumina, CA, USA). Raw reads (around 14M of reads per sample) were evaluated using FastQC v0.12.1 (2023–03–01) as post-sequencing Quality Control. Raw reads were filtered by Trimmomatic v0.39 (Bolger et al., 2014) with following parameters: ILLUMINACLIP: TruSeq3-PE.fa:2:30:10, LEADING:3, TRAILING:3, SLIDINGWINDOW:4:20, and MINLEN:36. A Phred quality score threshold of Q20 was applied for read filtering. The whole-genome shotgun sequences of the isolates generated in this study were deposited in GenBank under the BioProject accession number PRJNA1173090. The *de novo* assembly was performed by SPAdes Assembler v3.14.1 (2020–08–25) (Bankevich et al., 2012) on high-quality paired-ends reads for each isolate. Each genomic assembly contained contigs longer than 200 bp according to NCBI instructions (https://www.ncbi.nlm.nih.gov/genbank/wgsfaq/). ARGs were searched with AMRFinder Pathogen Detection (VERSION: 3.12.8, 2024-01-31.1). Multilocus Sequence Typing (MLST) with MLST Pasteur scheme (VERSION: 2024-03-09) (https://github.com/tseemann/mlst; accessed on March 2024). This scheme was selected for its high discriminatory power in *A. baumannii*, ability to provide stable sequence type (ST) assignments, and reliability in tracking clonal lineages across different hospitals and collection years. Additionally, it ensures comparability with previous epidemiological studies. DNA sequences of bacterial Virulence Factors (VFs) were retrieved from the Virulence Factors Database (VFDB). Virulence markers were searched with a minimal coverage of > 90% and an e-value cut off of 0.0000001 Analyses of the contigs sequences for each sample were performed using blastn v2.11.0+ (2020–10–11). Sequence analyses were performed using a commercial service (Bio-Fab Research srl, Roma, Italy).

### Biofilm formation assay

Biofilm formation was assessed using the microtiter plate assay and categorized as biofilm-forming producers as previously described (Ambrosi et al., 2017). Three independent experiments were performed.

### Desiccation tolerance assay

Overnight cultures were adjusted to OD_600_ ≈ 1.0, harvested by centrifugation, and washed twice with 2 ml of sterile water. Cell suspensions (10 µL) were deposited into a 96-well polystyrene microplate in triplicate and air-dried for 2–3 h in a biosafety cabinet until they were visibly dry. Uncovered plates were then placed in an airtight transparent plastic bag with silica beads and incubated for up to six days at 25°C with a relative humidity of 25 ± 2%. After incubation, the bacteria were rehydrated with water, gently scraped, plates rocked for 30 min to collect all cells from the bottom of the microplates, serially diluted in PBS and plated onto LB agar containing ampicillin (100 µg/ml). The percentage survival was calculated as:


$$\%\text{ survival}=\frac{\text{final}\;\text{CFU/ml}}{\text{initial}\;\text{CFU/ml}}\times 100$$

### Surfaced-associated motility, protease, and hemolytic activities

Surface-associated motility was evaluated by spotting 5 µL of an overnight culture on freshly prepared soft agar plates incubated at 37°C for 24 h., as previously described (Scribano et al., 2024). The motile clinical strains AB5075 and ATCC 17978, as well as the not motile ATCC 19606 were included as controls. Protease activity was assessed by spotting 5 µL on LB agar containing 1% skim milk. *Pseudomonas aeruginosa* PAO1 was included as the positive control. One microliter of the overnight culture was spotted onto Columbia agar plates (Becton Dickinson™, Milan, Italy) and incubated for 24 h to assess hemolytic activity and macrocolony types.

### *In vitro* infection

The human A549 lung epithelial cell line type II (ATCC CCL185, lab collection) and bladder cell line 5637 HTB-9 (ATCC-LGC, Milan, Italy) were cultured in DMEM supplemented with 10% fetal bovine serum (FBS), 1% glutamine, 1% penicillin-streptomycin, and 50 µg/ml gentamycin. Cell monolayers on 35 mm culture dishes at a density of 2.5 x 10^5^/ml were infected with clinical isolates at a multiplicity of infection (MOI) of 10 and incubated at 37°C with 5% CO_2_ for 2.5 h before adding colistin (10 µg/ml, BioChemica) to kill extracellular bacteria. After 48 h, cell monolayers were washed five times with PBS and lysed with 1 ml 0.1% Triton X-100. The recovered bacteria were serially diluted and plated onto LA plates to determine the number of colony-forming units (CFU)/ml.

### Statistical analyses

GraphPad Prism Software (10.3.1) was used for statistical analyses. Normality was assessed using the Shapiro-Wilk test or Kolmogorov-Smirnov test. Phenotypic characteristics were analyzed using one-way ANOVA, and followed by Dunnett’s *post hoc* test for multiple comparisons. Pearson correlation was used to assess the relationships among phenotypic traits, with data transformed using the log (Y) function prior to analysis. To evaluate whether the number of antibiotic resistance genes (ARGs) influence MIC values, the same analysis was performed. Statistical significance for all tests was set at P< 0.05. Moreover, **Supplementary Figures S1**, **S2** were generated with Microsoft Excel and GraphPad Prism, respectively.

### Data availability

The data presented in the study are deposited in the NCBI GenBank repository, accession numbers PRJNA1173090.

## Results

### Antibiogram profiles and genomic content of ARGs

The minimal inhibition concentration (MIC) was assessed for all isolates, and values were interpreted according to EUCAST 2023 criteria (**Table 2**). Based on MIC values, all isolates were classified as extensively drug-resistant (XDR). Apart from carbapenem-resistance, few isolates (7/30) were susceptible to amikacin, whereas only one to tobramycin (**Table 2**). All isolates were susceptible to colistin. Overall, strains collected from urine were more resistant compared with respiratory isolates. EUCAST was chosen over clinical and laboratory standard institute (CLSI) as it is the standard in Europe, and provides continuously updated breakpoints based on pharmacokinetic and clinical data, ensuring a more precise and stringent classification of resistance, particularly for carbapenems. To associate the clinical breakpoints to genotypic data, the genome of each isolate was extracted and sequenced. According to the Pasteur scheme, all *A. baumannii* isolates belonged to ST2; but one, isolate BO29, belonging to ST632 (Ambrosi et al., 2016).

**Figure 1:**
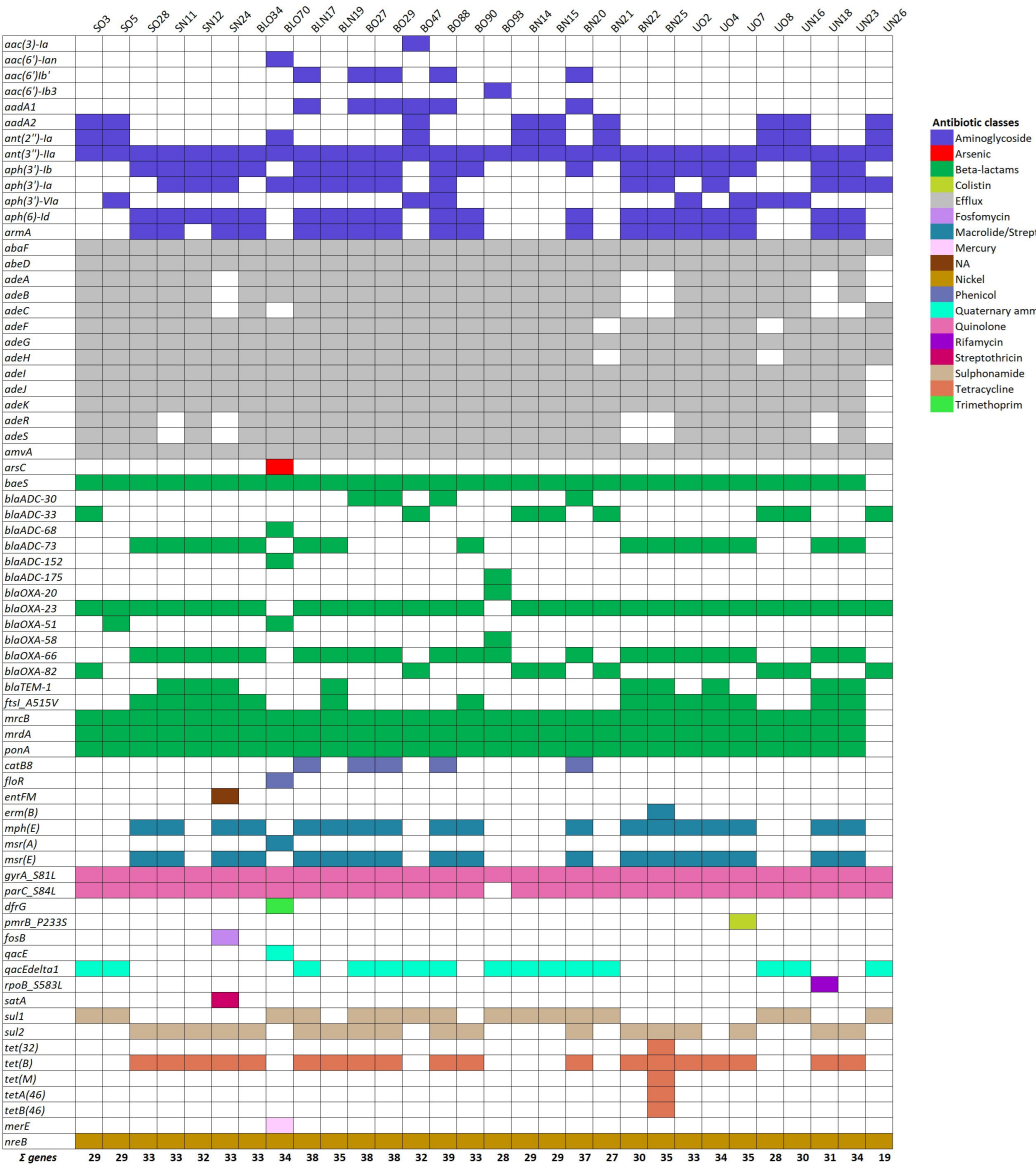
Genes directly and indirectly related to AR mechanisms detected in the isolate collection. Heat map of ARGs identified using whole genome sequence analysis. Color-code represents genes involved in the resistance of the different classes of antibiotics, heavy metals, and disinfectants, as indicated. NA, not applicable.

Genes directly and indirectly related to AR mechanisms were identified (**Figure 1**). Statistical analysis revealed a significant difference in the number of ARGs among isolates. On average, 46% (33/71) of conserved genes were harbored by older isolates, with older respiratory isolates exhibiting a 12.5% increase ARGs number compared to recent urine isolates (*P* = 0.02). Regarding the genes conferring resistance to aminoglycosides, 13 genes were found in the genomes’ isolates (**Figure 1**), thereby accounting for the high levels of resistance found in the MIC data (**Table 2**). Notably, the number of aminoglycoside resistance genes showed a strong positive correlation with the MIC of gentamicin (r = 0.43, *P* = 0.02) and amikacin (r = 0.70, *P* = 0.00002). Among these, the *ant (3*)*-IIa* variant was present in all isolates, followed by *aph(3’)-Ib* and *aph (6*)*-Id* (19/30), *armA* (18/19)*, aph(3’)-Ia* (15/30), *ant(2’)-Ia* (10/30)*, aadA2* (9/30)*, aph(3’)-Via* (7/30)*, aadA1* (6/30)*, aac (6*)*-Ib’* (5/30)*; aac (3*)*-Ia*, *aac(6’)-Ian*, and *aac (6*)*-Ib3* were found in single isolates. Interestingly, the overall content of aminoglycoside resistant genes was found less in recently isolated strains in comparison to old ones, particularly for urinary strains (**Figure 1**).

Among class D OXA-type carbapenemases, the *bla_OXA_*_-23_ gene was present in the vast majority of isolates (93%, 28/30) (**Figure 1**). Interestingly, the *bla_OXA_*_-51_-like carbapenemase gene, including *bla_OXA_*_-66_ and *bla_OXA_*_-82_ variants, was carried by all isolates. Conversely, the *bla_OXA_*_-58_ and *bla_OXA-20_* genes were carried by one strain each (3%, 1/30), and the class A gene, *bla_TEM-1_* was found in 30% of isolates (9/30) (**Figure 1**). Several intrinsic *Acinetobacter*-derived cephalosporinases (ADCs) genes belonging to class C were found in the genomes of isolates (**Figure 1**). The most prevalent variants were *bla_ADC-73_* (50%, 15/30), *bla_ADC-33_* (27%, 8/30), and *bla_ADC-30_* (13%, 4/30) (**Figure 1**). Other variants such as *bla_ADC-152_*, and *bla_ADC-175_* were identified only in old isolates BO70, and BO93, respectively (**Figure 1**). It was also identified the mutation A515V in the penicillin-binding protein 3, FtsI, an essential division component in fourteen isolates, which is associated with an increase in meropenem MIC values (Taguchi et al., 2019). From our data, the overall content of beta-lactamase and carbapenemase genes seems increased in recently collected strains from respiratory specimens, while it is stable among urinary isolates (**Supplementary Figure S1A**).

Moreover, the *parC_S84L* and *gyrA_S81L* genes conferring resistance to quinolones were found in 97 and 100% of the isolates, respectively (**Figure 1**). The *sul1* and *sul2* genes involved in sulfonamide resistance were less present, being both found in 53% of the genomes (16/30). The overall content of quinolone and sulfonamide resistance genes appears to have increased and decreased in recent respiratory strains, respectively. Conversely, while the content of quinolone resistance genes remains the same in both old and new urinary strains, there is an increase in sulfonamide resistance genes in recent urinary strains (**Figure 1**). Furthermore, five genes associated with tetracycline resistance mechanisms were identified, *tet(B), tet(M), tet (32*)*, tetA (46*), and *tetB (46*), showing higher prevalence in recent respiratory isolates and lower prevalence in recent urinary strains (**Figure 1**). The *tet(B)* gene, an efflux pump (belonging to the major facilitator superfamily [MFS] superfamily) known to mediate resistance to tetracycline, doxycycline, and minocycline, was the most prevalent among our strain collection, accounting for 67% (20/30). The other tetracycline resistance genes, including *tet (32*), *tet(M)*, conferring resistance to tetracycline by ribosomal protection, and *tetAB (46*), encoding a predicted heterodimeric ABC transporter was only described in *Streptococcus australis* (Warburton et al., 2013) were found only in strain BN25. To assess the contribution of these tetracycline resistance genes, we determined the MIC of tetracycline. BN25, BLN17, and UO7 exhibited MICs greater than 256 µg/ml, while other strains had MICs >16 µg/ml, highlighting the key role of Tet efflux pumps, including *tetB* (Huys et al., 2005).

Moreover, all isolates were colistin-susceptible, according to EUCAST breakpoints (https://www.eucast.org/). Notably, isolate UO7 carried the P233S mutation in the histidine kinase encoded by the *pmrB* gene, described as a key mutation for conferring resistance to polymyxins under certain conditions, accounting for the higher MIC value (Sun et al., 2020; Hahm et al., 2022). Although macrolide resistance was not tested, the *A. baumannii* common *mph(E)-msr(E)* operon was found in 57% of isolates (17/30). Differently, the *erm(B)*, and *msrA* genes were individually found in only two isolates, BN25 and BO70, respectively (**Figure 1**). Overall, macrolide resistance gene content was found increased in recent respiratory isolates, and decreased in recent urinary strains (**Supplementary Figure S1**). Furthermore, the *catB8* gene, encoding a chloramphenicol acetyltransferases protein type B (Liao et al., 2024), was identified in 17% (5/30) of isolates. Differently, the *floR* gene, conferring resistance to florfenicol and chloramphenicol, was found only in one isolate (**Figure 1**).

Several genes encoding multidrug efflux pumps were identified; the most prevalent ones were *amvA* and *abaF*, encoding MFS pumps, which were found in all isolates (Sharma et al., 2016; Short et al., 2021) (**Figure 1**). Differently, the *adeC* gene, encoding the outer membrane protein of the three-component efflux machinery of the resistance nodulation division (RND)-type superfamily (Salehi et al., 2021), was found in 80% of isolates (24/30). Interestingly, the mutation P116L in the regulatory gene *adeR* associated with AdeAB overexpression was identified in only one isolate (1/30), UO7 (**Figure 1**) (Marchand et al., 2004). The RND efflux pump *adeFGH* were highly present in all isolates, ranging from 100% for adeG to 97% (29/30) for *adeF* and *adeH* (**Figure 1**). In addition, the efflux systems involving *qac* genes (*qacE* and *qacE* delta1) that confers resistance to biocides were present in 3 (1/30) and 50% (15/30) of isolates, respectively (Shafaati et al., 2016). The nickel/cobalt tolerance gene *nreB* was found in all isolates (Neidhöfer et al., 2023). Overall, we found a slight decrease in the content of efflux pump genes in both groups of recent strains (**Figure 1**; **Supplementary Figure S1**). Other genes involved in resistance to heavy metal efflux (*arsC*, and *merE*), fosfomycin (*abaF* and *fosB*), trimethoprim (*dfrG)*, rifamycin (*rpoB_S583L)*, and streptothricin *(satA)*, were the least prevalent among isolates (**Figure 1**). The highest content of ARGs was found in strain BO88 (39 genes), followed by strains BN17, BO29, and BO27 (Neidhöfer et al., 2023). According to the antibiogram profile, the most common genotype in strains displaying the highest MIC values was: *ant(3’’)-IIa, abaF, abeD, adeABFGHJK, amvA, baeS, bla_OXA-23_, bla_OXA-51_* (variants included*), mrcB, mrdA, ponA, gyrA_S81L, nreB, parC_S84L.*

### Growth rates and genomic content of key metabolic genes

**Figure 2:**
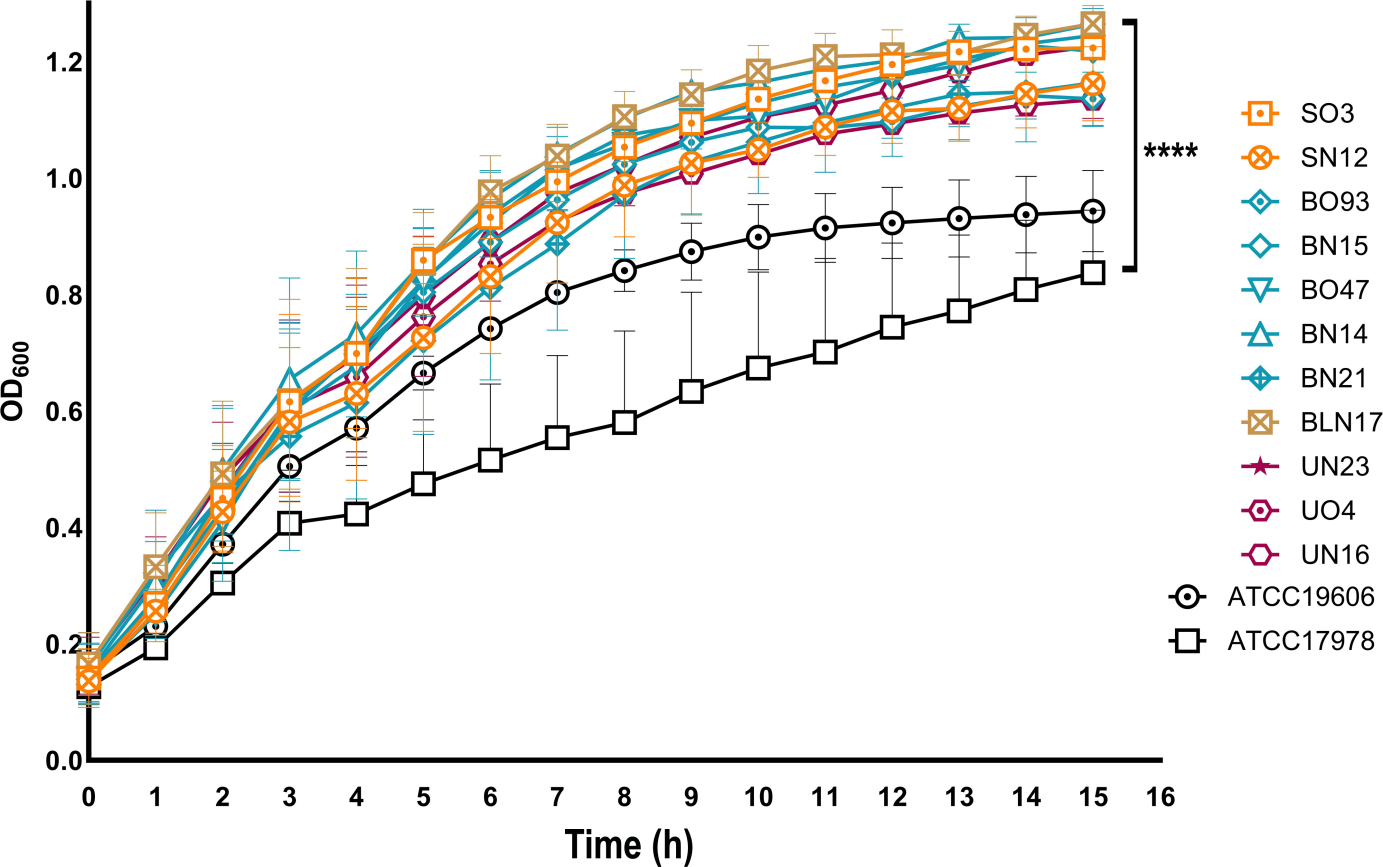
Growth rates of the clinical isolates. Overnight cultures were diluted to 1:100 in LB in a 96-wells microplate. The OD_600_ was determined every 30 min using a plate reader (BioTek SynergyHT). Only the growth curves of the 11 fastest-growing isolates, compared to both reference strains, are presented. Means and standard deviations were obtained from three independent experiments. ****P< 0.0001.

**Figure 3:**
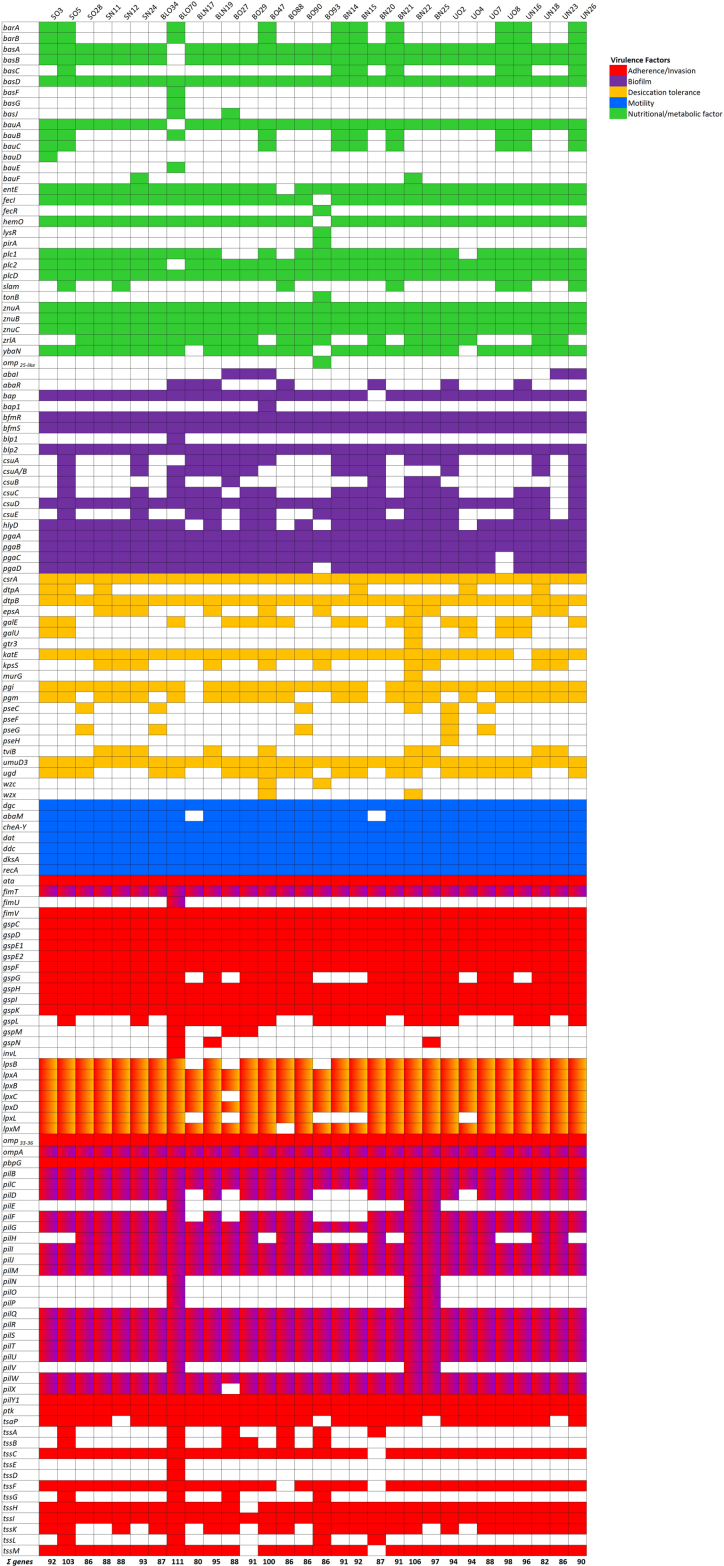
Genes directly and indirectly related to virulence detected in the isolate collection. Heat map of virulence genes identified using whole genome sequence analysis. Color-code represents genes involved in different aspects of virulence, adhesion, biofilm, secretion systems, exotoxins, immune modulation, and nutritional/metabolic factors, as indicated.

The growth properties of the isolates were evaluated. The two reference strains ATCC 17978 and ATCC 19606 were included as controls. As shown in **Figure 2**, eleven isolates collected from 2022, and mainly isolated from the respiratory tract (7/11), showed a significant faster growth in comparison to both reference strains (*P*< 0.0001). Key genes related to *A. baumannii* metal ion metabolism uptake systems were analyzed (**Figure 3**), including the *bar/bas/bau/entE* (synthesis, secretion, and uptake acinetobactin/enterobactin, *pbpG* (penicillin-binding protein 7 (PBP7), *fecIR/hemO/tonB* (hemin or hemoglobin), *pirA/slam/ybaN* (transporters), *znuABC* (zinc acquisition system), *zrlA* (zinc peptidase), *plc1/plc2/plcD* (phospholipases), and *lysR* (transcriptional regulator) (Sheldon and Skaar, 2020; Bateman et al., 2021; Cook-Libin et al., 2022); *basD, plcD*, and *znuABC* were conserved in all isolates, while prevalent *basAB, bauA, entE, fecI, hemO*, and *plc2* (29/30, 97%) (**Figure 3**). According to growth rates, the most common genotype in fastest-growing isolates was: *basABD, bauA, entE, plc2, plcD, znuABC* (**Figures 2**, **3**).

### Biofilm-forming activity, desiccation tolerance and genomic content of related genes

**Figure 4:**
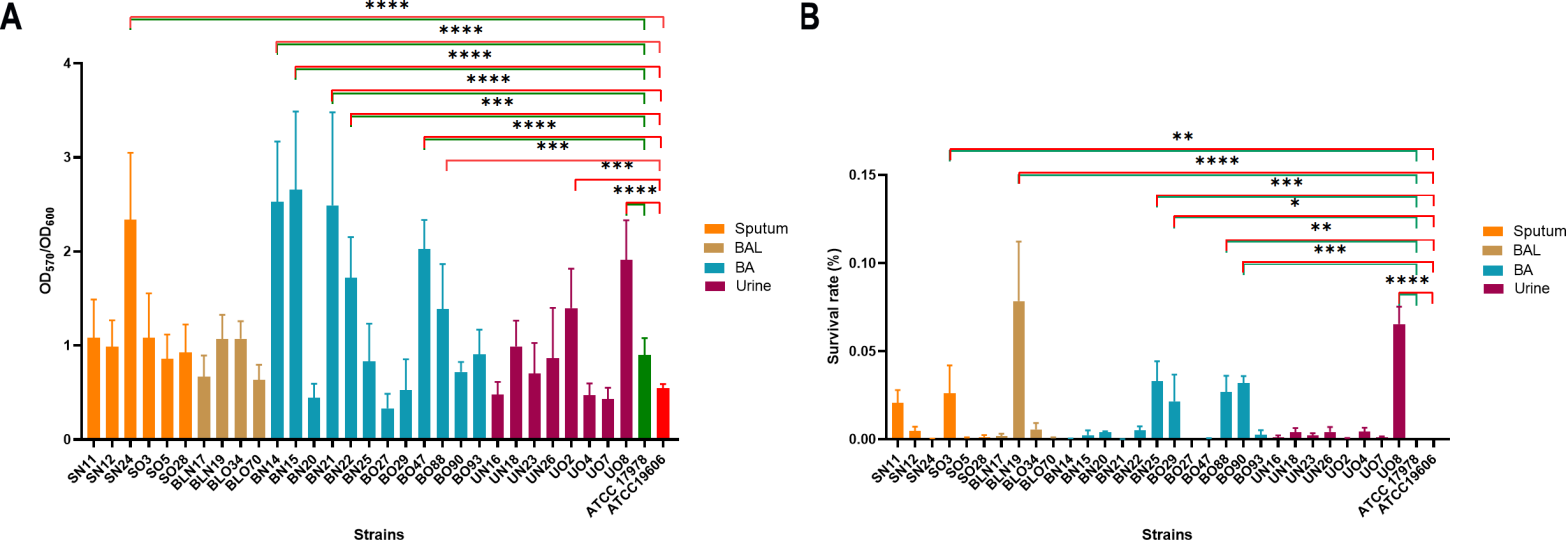
Ability of *A. baumannii* isolates to form biofilms and tolerate desiccation conditions. **(A)** *A. baumannii* strains were inoculated on polystyrene plates. After 48 h, quantification of biofilm formation was measured by the Crystal violet method (Ambrosi et al., 2017). Data are means ± standard deviation from at least three independent experiments, eight wells per strain (n=24). **(B)** Approximately 10^7–^10^9^ CFU/strain were inoculated in a 96 microplate. Plates were kept at 25°C with a relative humidity (RH) of 25 ± 3% for 6 days. Each isolate was recovered from three wells. Data are the ratio between the final and initial CFU/ml. Three independent experiments were performed in duplicate (n=6). Asterisks represent p values evaluated by one-way ANOVA in comparison to ATCC 17978 (green) and to ATCC 19606 (red); *P<0.05, **P<0.01, ***P<0.001, ****P<0.0001.

The ability of *A. baumannii* clinical isolates to form biofilms represent crucial features in clinical settings. Therefore, the biofilm-forming ability on polystyrene microtiter plates of each isolate was measured by Crystal Violet staining (**Figure 4**). Isolates arbitrarily grouped according to A_570_/A_600_ values as weak (<0. 47), moderate (0.47-0.70), and strong biofilm producers (>0.70) accounted for 16.7% (5/30), moderate for 23.3% (7/30), and strong for 60.0% (18/30), respectively (**Figure 4A**). Nine isolates (SN24, BO47, BO88, BN15, BN14, BN21, BN22, UO2, and UO8) showed significant differences in biofilm formation compared with both reference strains, with the majority belonging to the recently collected BA specimens (71.4%, 5/7). Isolates BO88 and UO2 were significant only in comparison with ATCC 19606 reference strain (**Figure 4A**). Interestingly, we found a significant positive correlation between biofilm producer strains and amikacin resistance (*r* = 0.43, *P* = 0.016). Several genes that contribute to biofilm formation were searched, including *bap/bap*-like proteins (biofilm associate proteins), *csuA-E* (chaperone-usher type I pili), *pil* and *fim* genes (type 4 pili), *pgaA-D* genes (poly-β-1,6-N-acetylglucosamine), *bfmRS* (the two-component system), *ompA* (the major Gram-negative porin), *hlyD* (Type 1 Secretion System) and *abaIRM* (quorum sensing). All isolates carried the *bpl2*, *bfmRS*, *ompA*, *csuD*, *pgaAB*, *pilBCGIJMQRSTUWY1*, and *fimTV* genes (**Figure 3**). Interestingly, the *bap* gene was identified in all but one (the BN20 isolate), while *bap1* and *bpl1* were identified only in BO47 and BO70, respectively (**Figure 3**). According to the biofilm results, the genes shared among all the strains displaying the strongest biofilm-forming ability were: *bap/blp2/bfmRS/csuD/pgaAB/fimTV/ompA/pilBCGIJlMlQRSTUWXY1* (9/9), with a prevalence of *hlyD*, *pgaCD* (8/9). Unexpectedly, the *abaIR* genes were found in a minority of strains, UN16, BLN17, BLN19, BLO70, and UO2, the latter only displaying a strong biofilm-forming activity (**Figure 3**). Conversely, the *abaR* gene, without *abaI*, was found only in BN20 and B088. Additionally, we found significant positive correlation between fast-growing and strong biofilm-forming isolates (*r* = 0.42, *P* = 0.022) (**Supplementary Figure S2**). Another critical feature for healthcare settings is *A. baumannii* desiccation tolerance since it allows long term survival, and therefore increases bacterial spread in the hospital environment (Farrow et al., 2018; Bashiri et al., 2021). Therefore, the tolerance to desiccation for 6 days was tested. Starting with an initial number of bacteria (approximately 10^8–^10^9^ CFU/ml/strain), the number of recovered bacteria varied (approximately 10^1–^10^4^ CFU/ml/strain) after six days of dryness and 25% of relative humidity. Seven isolates, SO3, BLN19, BN25, BO29, BO88, BO90, and UO8, displayed significant differences in their desiccation tolerance (**Figure 4B**). No reference strains were recovered. Among desiccation-tolerant strains, 57.1% (4/7) respiratory and 14.3% (1/7) urinary isolates, respectively, were retrieved before 2013. Genes directly and indirectly related to desiccation tolerance were searched, including *bfmRS, csrA* (carbon storage regulator A), *epsA* (exopolysaccharide), *katE* (catalase), *recA* (main recombinase), *murG* (peptidoglycan)*, lpx/lpsB* (lipopolysaccharide), *wzc/wzx/gtr3/kpsS/pgm/pse* (capsule), *galU/ugd/pgi/pgm* (sugar precursors), *dtpAB* (hydrophilins), *umuCD* (error-prone DNA polymerase V), *adeABC* and *adeIJK* (efflux pumps*)* genes (Tipton et al., 2018; Singh et al., 2019; Green et al., 2022) (**Figures 1**, **3**). Genes conserved in all isolates were *csrA, dtpB, umuD3, lpxABD, recA* and *adeG*, while prevalent *pgm, katE, lpxCM*, and *adeIJK*, (29/30, 97%) (**Figure 3**). According to the desiccation results, the most common genotype among the strains displaying the highest desiccation tolerance was: *bfmRS/csrA/katE*/*lpxABCD/pgi/dtpB/umuD3/adeAB/adeIJK.*

### Surface-associated motility and genomic content of related genes

**Figure 5:**
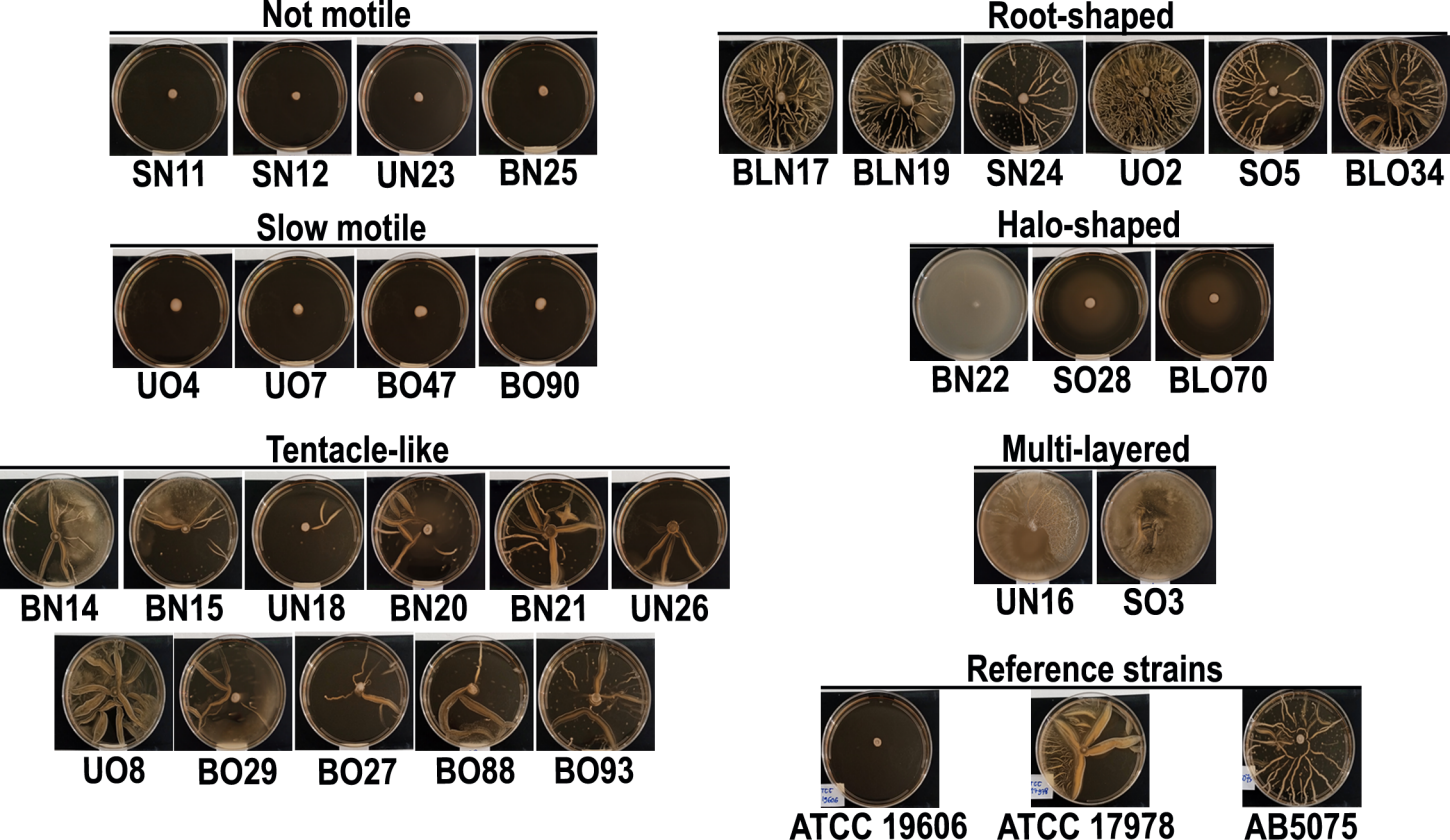
Surface-associated motility of clinical isolates. Each isolate was spotted on top of a soft agar plate. After 24 h of incubation at 37°C, plates were photographed. Three independent experiments were performed in duplicate (n=6). Representative images are shown. Reference strain AB5075 was included in the experiment for its different shape of surface-associated motility.

*A. baumannii* can move via surface-associated motility, an appendage-independent kind of movement (Jeong et al., 2024). Hence, the surface-associated motility of isolates was tested on soft agar plates. Isolates SN11, SN12, UN23, and BN25 displayed a not motile phenotype, while UO4, UO7, BO47, and BO90 a very slow motility (**Figure 5**). Conversely, the other isolates displayed surface-associated motility characterized by peculiar shapes, including tentacle-like (37%, 11/30), root-shaped (20%, 6/30), halo-shaped (10%, 3/30), and multi-layered (2/30) (**Figure 5**). Notably, the different patterns of motility were equally distributed between past and current isolates. Several genes have been shown to be involved in surface-associated motility, including *ddc/dat* (production of 1,3-diaminopropane), *dgc* (diguanylate cyclase), *cheA/Y* (two component system), *dksA* (DnaK suppressor protein), *abaIRM*, *recA*, *ompA* (Jeong et al., 2024). Interestingly, all the aforementioned genes were conserved in all isolates with the exception of *abaM* gene that was not found in BNL17 and BN20 (28/30, 93%) (**Figure 3**). According to the surface-associated motility, the most common genotype of motile isolates was: *dgc, cheA-Y, dat, ddc, dksA* and *recA.*

### Host cell line invasiveness and genomic content of related genes

**Figure 6:**
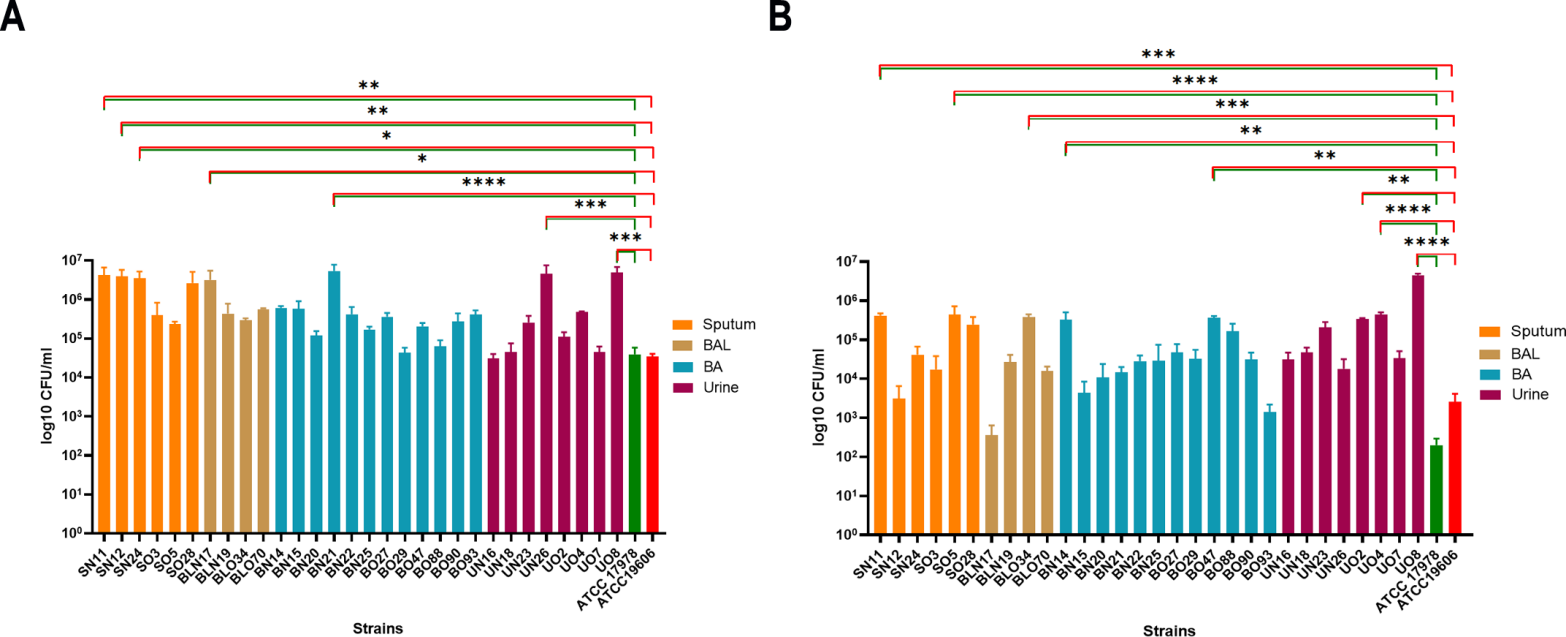
Ability of clinical isolates to be internalized in lung and bladder epithelial cells. Semi-confluent A549 **(A)** and HTB-9 **(B)** cell monolayers were infected with each isolate or ATCC 17978 or ATCC 19606 reference strains using a multiplicity of infection (MOI) of 10. Uninfected cells were used as control. After 2 h of adhesion, the medium was supplemented with colistin (10 µg/ml) to kill extracellular bacteria. Intracellular bacteria were enumerated after additional 48-h of incubation. Data (CFU/ml) are means ± standard deviation from at least five independent experiments, in duplicate (n=10). Asterisks represent p values evaluated by one-way ANOVA in comparison to ATCC 17978 (green) and to ATCC 19606 (red); *P<0.05, **P<0.01, ***P<0.001, ****P<0.0001.

Some *A. baumannii* clinical isolates can enter epithelial cells to evade host immune responses and limit antibiotic accessibility (Ambrosi et al., 2020; Rubio et al., 2022). Therefore, infection experiments using human lung and bladder epithelial cells were conducted to quantify the number of intracellular bacteria after 48 hours (**Figure 6**). Compared to the two reference strains, ATCC 17978 and ATCC 19606, a significantly higher invasive capacity in lung epithelial cells was observed in 23% (7/30) of the tested strains, most of which were retrieved from respiratory specimens (5/7, 71%), reaching values greater than 3 × 10^6^ CFU/ml (**Figure 6A**). Additionally, the majority of these invasive strains were recently isolated (6/7, 86%). The same experiment with bladder epithelial cells revealed that 27% of the analyzed strains could invade this host cell line (8/30). In this experimental set, eight isolates demonstrated greater invasiveness compared to both reference strains, 75% (6/8) of these were old strains and 38% (3/8) were retrieved from the urinary tract (**Figure 6B**). Intriguingly, among invasive strains, SN11, and UO8 were able to invade both host epithelial models, suggesting a cell-type non-specific invasion phenotype (**Figure 6**). Genes directly and indirectly related to invasiveness were searched (**Figure 3**), including *ata* (*Acinetobacter* trimeric autotransporter), *gsp* (T2SS), *tss* (T6SS), *invL* (adhesin), *pbpG*, *lps*, *omp33-36/ompA*, *plc*, *pil* genes. According to the invasion experiments, the most common genotype displayed by invasive isolates was: *ata, fimTV, gspCDE1E2FHIK, lpsABCDM, omp33-36, ompA, pbpG, pilBCGIJMQRSTUWXY1, ptk*, and *tssCFHIM* (**Figure 3**). Unexpectedly, *invL* was found only in BO70 (**Figure 3**). No major genetic differences between the cell-type non-specific invasion phenotypes and the other invasive isolates were found (**Figure 3**). Notably, significant negative correlations were found between urinary (*r* = -0.44, *P* = 0.014) and recent isolates (*r* = -0.36, *P* = 0.048) and the invasiveness of lung and bladder epithelial cells, respectively. Moreover, we found significant positive correlation (*r* = 0.40, *P* = 0.030) between strong biofilm-forming isolates and invasiveness of A549 cells (**Supplementary Figure S2**).

### Proteolytic and hemolytic activities and genomic content of related genes

**Figure 7:**
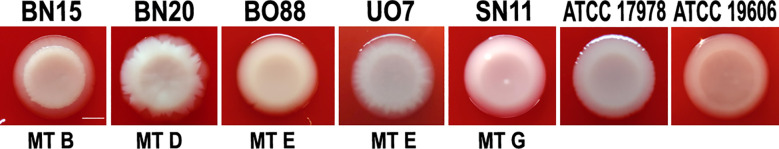
Macrocolony types (MTs) of clinical isolates on blood agar plates. One µl of each isolate was spotted on Columbia Agar with 5% sheep blood. After 24 h, macrocolonies were photographed. Representative images of one representative isolate per different MTs. Scale bar, 1 cm.

As an additional assay of virulence traits, protease and hemolytic activities were assayed on skim milk and blood agar plates, respectively. No proteolytic and hemolytic activities could be detected in the clinical isolates in comparison with *Pseudomonas aeruginosa* PAO1 and *Escherichia coli* H20P used as positive controls (Ambrosi et al., 2019). The *hlyD* gene, reported to be a putative hemolysis-related gene (Schramm et al., 2019), was found in 83% (25/30) of the isolates (**Figure 3**). Additionally, among the *plc* genes (Fiester et al., 2016), *plc1* was absent only in isolates BO29 and UO4, while *plc2* was absent only in BO70 (**Figure 3**). Conversely, *plcD* was common to all isolates (**Figure 3**). We noticed a high degree of heterogeneity among the clinical isolates in the shape of the macrocolonies (Valcek et al., 2023); in particular, four previously reported and one new macrocolony types (MTs) were identified (Valcek et al., 2023) (**Figure 7**). This new macrocolony shape (MT G), shared by 50% of the strains (15/30), was characterized by a deep opaque center expanding to the round edge (**Figure 7**; **Table 3**). Interestingly, both *plc2* and *plcD* were shared among strains displaying MT G (**Figure 3**). No morphology resembling types A and C was observed in any of our isolates (Valcek et al., 2023). The second prevalent MT was MT E, followed by MT F; only one isolate had a MT B (BN15), and one MT D (BN20) (**Figure 7**; **Table 3**). This MT pattern was homogeneously shared between past and recent isolates (8 and 7, respectively). Additionally, no correlation between MTs and surface-associated motility patterns was found (**Figures 5**, **7**; **Supplementary Figure S2**) (*r* = 0.080, *P* = 0.256).

**Table 3:** Macrocolony types (MTs) (Valcek et al., 2023).

MTs	Isolates
A	–
B	BN15
C	–
D	BN20
E	SO5, BLN17, BLO70, BO88, BN22, BO27, BN25, BO90, UO2, UN16, ATCC 17978
F	BN14, UO7, UN18
G	SO3, SO28, SN11, SN12, SN24, BLO34, BO47, BO93, BO29, BN21, BLN19, UN23, UO4, UO8, UN26, ATCC 19606

## Discussion

From an evolutionary perspective, *A. baumannii* demonstrates remarkable adaptability, acquiring a wide range of virulence factors through high genomic plasticity. Understanding how this pathogen evolves to gain virulence and AR is critical for developing strategies to combat this public health threat. Thirty clinical isolates, 22 respiratory, and 8 urinary, were analyzed. The proportion of strains aligns with previous reports, where respiratory isolates dominate (39.5%) over uropathogenic isolates ranging from 6.1% to 29.3% (Bagińska et al., 2021). In line with current reports, the ST2 was the most represented in our collection (Hansen et al., 2023; Müller et al., 2023). All the isolates displayed an XDR phenotype, with a high number of ARGs in each genome (**Table 2**; **Figure 1**). Among the 71 identified ARGs, old isolates have a higher gene content (> 30) with respect to recent ones (27 vs. –19 genes, respectively), particularly old respiratory isolates exhibited 12.5% increases in the average number of ARGs compared to recent urine isolates (*P* = 0.02) (**Figure 1**; **Supplementary Figure S1A**). Vice versa, recent respiratory isolates carried an overall higher content of resistance genes for beta-lactams, carbapenems, quinolones, and tetracyclines, compared with old ones (**Figure 1**). Increased resistance to quinolones and carbapenems is in accordance with the ECDC report of invasive strains (https://atlas.ecdc.europa.eu/public/index.aspx). Furthermore, the number of aminoglycoside resistance genes positively correlated with the MIC of gentamicin (r = 0.43, *P* = 0.02) and amikacin (r = 0.70, *P* = 0.00002), supporting their role in conferring resistance. Notably, recent respiratory isolates exhibited lower amikacin MICs, likely due to the reduced prevalence of aminoglycoside resistance genes (**Table 2**; **Figure 1**). In the past, the synergistic effects of combined treatment with aminoglycosides and carbapenems or colistin for *A. baumannii* infections were exploited (Zhang et al., 2023). Hence, the lower prevalence of aminoglycoside resistance genes in our recent strains may reflect the decline in the use of this therapeutic approach, given its lack of significant improvement in clinical outcomes (Shields et al., 2023).

The lower content of macrolide resistance genes in recent isolates may be linked to the WHO’s 2017 recommendation against macrolides (www.iris.who.int, **Figure 1**). The genetic adjustments made by recent isolates over old ones might be due to lower contacts with donor species and/or that global and local antibiotic guidelines changes for carbapenem-resistant *A. baumannii* (Kim et al., 2021). Notably, all isolates remained susceptible to colistin; however, two isolates (SO5 and UO7) had MICs near the resistance breakpoint (EUCAST 2023), highlighting the presence of two colistin-heteroresistant strains. EUCAST clinical breakpoints were used for antibiotic susceptibility testing, as they are standard in Europe and facilitate interlaboratory comparisons; however, it is worth mentioning that, although EUCAST and CLSI breakpoints are aligned for carbapenem-resistant *A. baumannii*, notable discrepancies between the two methodologies exist for specific aminoglycoside and tetracycline subclasses, which should be duly considered (Farshadzadeh et al., 2018; Yang et al., 2019; Bulens et al., 2024; Halim et al., 2024). Moreover, the content of genes encoding efflux pumps was stable among isolates. While the *adeE* gene, typically of *Acinetobacter nosocomialis*, was missing, the majority of isolates (25/30) carried the *adeB* gene, which was found shared among bacteria belonging to the *Acinetobacter* genus (Hou et al., 2012; Cosgaya et al., 2019).

Distinctive traits of recent isolates include faster growth (*P*< 0.0001), a partial association with MT G (7/11), an average of 16.9 nutritionally-related genes, a strong biofilm-forming activity, and a lower desiccation tolerance than older ones (**Figures 2**–**4A**). These findings recall the behavior of multi-drug resistant (MDR) *A. baumannii* environmental isolates, in which this phenotype comes with a fitness cost that causes a significantly decrease in desiccation tolerance even with strong biofilm activity (Greene et al., 2016). Being these clinical isolates, a possible explanation is that these strains experienced less desiccation stress pressures in their growth environment Seven isolates, mostly from the past respiratory collection, survived six days of desiccation with an average survival rate of 3.44% (**Figure 4B**). Among the 21 desiccation-related genes identified, these isolates carried an average of 8.6 genes (**Figure 3**). Notably, the BfmR-dependent hydrophilin *dtpB* gene, involved in desiccation tolerance (Green et al., 2022), was found in all strains (**Figure 3**). However, only two strains, BO88 and UO8, retained both biofilm-forming and desiccation tolerance abilities (**Figures 3**, **4**). Desiccation tolerance was reported to be a typical characteristic of clinical *A. baumannii* strains (Hamidian et al., 2022). However, from our data, it seems that the hospital environment may not exert desiccation pressure, offering enough water or humidity for bacterial survival and growth, potentially selecting mainly for stronger biofilm-forming strains.

The positive correlation found between growth rate and biofilm formation (*r* = 0.42, *P* = 0.022) corroborates previous findings that biofilm development depends on bacterial growth rates (Farshadzadeh et al., 2018). Conversely, urinary strains, while having the highest nutritionally-related gene content, were not proficient biofilm formers (**Figure 4A**). In dominant and non-dominant *A. baumannii* STs, despite a very similar content and expression patterns of biofilm-related genes, such as *adeFGH, bap, csu, pga/b/c/d, abaR*, and *abaI*, the actual biofilm-forming activity was not significantly different (Kong et al., 2023). Strong biofilm-formers had an average of 12.3 biofilm genes (range: 8-15, **Figure 3**; **Supplementary Figure S1B**), and it seems that at least 8 genes are sufficient for proficient biofilm formation. Moreover, the positive correlation between strong biofilm producers and invasion of lung epithelial cells (*r* = 0.40, *P* = 0.030) strengthen the notion that biofilm matrix can effectively shield bacteria, allowing persistence, and, eventually, invasion host cells (Yang et al., 2019).

Surface-associated motility was observed in 26 isolates, with variability from slow to hypermotile phenotypes (**Figure 5**). Variability in surface-associated morphotypes suggests that efflux pumps, quorum-sensing molecules and surfactant-like compounds and proteins may influence motility and phenotypic diversity (Clemmer et al., 2011; Cosgaya et al., 2019; Pérez-Varela et al., 2019). Seven genes related to surface-associated motility were identified (**Supplementary Figure S1B**) (Blaschke et al., 2021; Corral et al., 2021; Jeong et al., 2024), but two isolates (BN20 and BLN17) lacked the *abaM* gene (**Figure 3**). The lack of surface-associated motility of isolate BLN17 could be due to the absence of genes encoding the AdeRS two-component system that regulates the RND efflux pump AdeABC, as previously described (De Silva and Kumar, 2018). Chemotaxis in *E. coli* is subject to a behavioral variability and phenotypic diversity (Waite et al., 2018); *E. coli* swimming depends on external signal processing as well as the proton motif force and the levels of individual expression of the involved proteins, known as potential modulators of chemotactic variability (Waite et al., 2018). Although *A. baumannii* lacks flagella, it is plausible that variability of surface-associated motility could be due to the to a combination of the activity of efflux pumps that, in turn, cause a variable secretion of surfactants, proteins or quorum sensing molecules (Clemmer et al., 2011; Pérez-Varela et al., 2019; Gaona et al., 2024).

Thirteen isolates demonstrated higher invasiveness into epithelial cells compared to reference strains (**Figure 6**). Recent respiratory isolates were more invasive in lung cells, while older isolates showed more invasiveness in bladder cells, with observed invasive colony numbers of 4 x 10^6^ and 1 x 10^6^, respectively (**Figure 6**). Interestingly, two strains, SN11 and UO8, exhibited a non-specific cell-type invasion phenotype, being capable of invading both cell lines (**Figure 6**). Overall, these results suggest a shift in recent respiratory strains toward lung tropism *in vitro*, reflecting their preferred colonization/infection site *in vivo* (Bagińska et al., 2021). Several bacterial pathogens exhibit host cell tropism, dependent on specific surface appendages/proteins that ensure the interaction between pathogens and their respective hosts, like type 1 fimbriae for uropathogenic *E. coli* (Sarshar et al., 2020). Among 63 conserved genes corresponded to invasiveness, 79% were across all isolates, encoding components of lipopolysaccharide (LPS), type II secretion system (T2SS), type IV secretion system (T4SS), and type VI secretion system (T6SS) (**Figure 3**; **Supplementary Figure S1B**). Several of these genes were shown to be involved in adhesion/invasion in *A. baumannii* (De Silva and Kumar, 2018; Ambrosi et al., 2020; Sarshar et al., 2021). Notably, the *invL* gene, encoding an invasin-like adhesin dependent on T2SS, involved in adhesion to urothelial cell cultures, was identified only strain BO47 (Jackson-Litteken et al., 2022) (**Figure 3**). Differently, the T2SS genes were found in all strains (*gspCDE1E2FHIK*), underlining the importance of this system for *A. baumannii* physiology and pathogenesis; indeed, T2SS contributes to *A. baumannii in vivo* fitness as well as protection to the human bactericidal activity (Johnson et al., 2016; Waack et al., 2017). Thus, other T2SS-dependent proteins might be involved in *A. baumannii* invasiveness; moreover, the high degree of conservation makes this an ideal target for innovative anti-*A. baumannii* therapeutics. It is worth mentioning that this study has some potential limitations, including a limited sample size, as well as geographical and time constraints. However, this study provides evidence of significant adaptations driven by clinical pressures. The findings herein reported could contribute to highlight key traits that could be targeted for improved infection control and treatment strategies.

**Figure 8:**
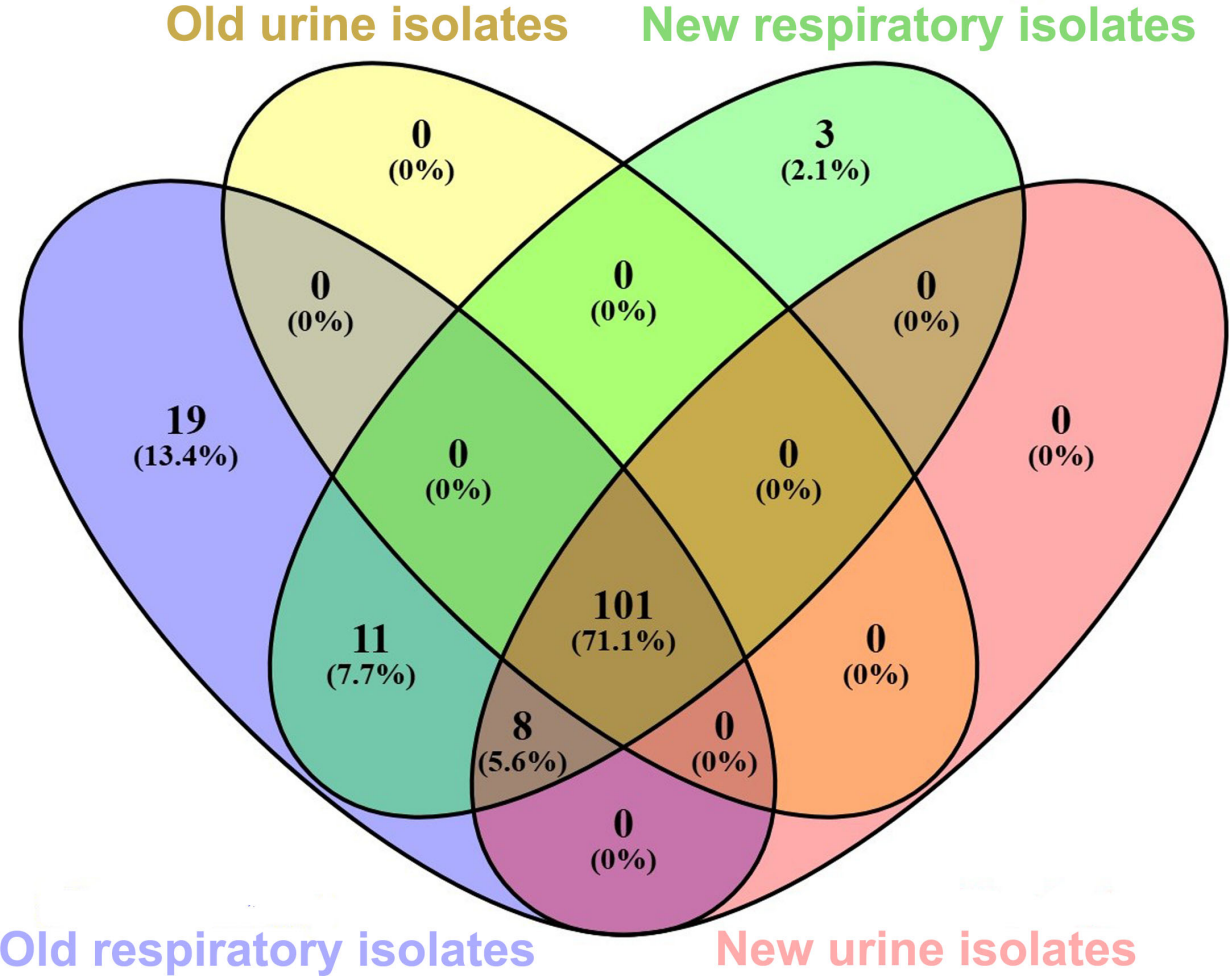
Venn diagram showing the number of common genes shared among the isolate collection. Each group is depicted by a different oval color, as indicated. Genes included are those reported in **Figures 1** and **3**. Numbers in the non-overlapping regions show the number of genes unique to that strain group. The given percentage was calculated by considering the total number of genes (142) as 100%. The graph was generated using Venn 2.1. online software.

In conclusion, significant genetic variability was observed between past and recent isolates, with 71.2% of identified genes distributed across all strains. Interestingly, 19 genes were unique to the old respiratory strains (**Figure 8**), mainly involved in adhesion and nutrient uptake (e.g. *fimU, gspM, tssBDEG, basFGJ, bauDE, bap1, wzc, pseG, lysR, fecR, pirA, tonB*, and *omp25-like*). The overlap between old and recent respiratory isolates was limited to 11 shared genes, including *pilE, pilNOPV, gspN, tssAL, tagX, pseC*, and *wzx*. Eight genes were conserved across recent urinary and respiratory strains (*tviB, kpsS, hemO, epsA, fecI, ybaN*, hlyD, and *slam*). Remarkably, no gene overlap was observed between past and recent urinary isolates, justifying the marked difference in the phenotypic traits. Old isolates were more desiccation-tolerant, and metabolically variable likely due to adaptation to broader nutrient sources and environmental conditions. In contrast, recent respiratory isolate exhibited increased growth, biofilm formation, and invasiveness, reflecting the dynamic evolution to clinical stress pressures. Overall, these findings support the genetic adaptation of *A. baumannii* in response to environmental pressures, including antibiotic use and host immune responses. Understanding these genetic changes will provide valuable insights for future therapeutic approaches and infection control strategies.

## Data Availability

The datasets presented in this study can be found in online repositories. The names of the repository/repositories and accession number(s) can be found below: https://www.ncbi.nlm.nih.gov/genbank/, PRJNA1173090.
