# Supplementary figures and images for: A decade of genomic and phenotypic adaptation of carbapenem-resistant Acinetobacter baumannii
Source: Front Cell Infect Microbiol. 2025 Apr 30;15:1527488. doi: 10.3389/fcimb.2025.1527488 (PMC12075148; doi:10.3389/fcimb.2025.1527488)

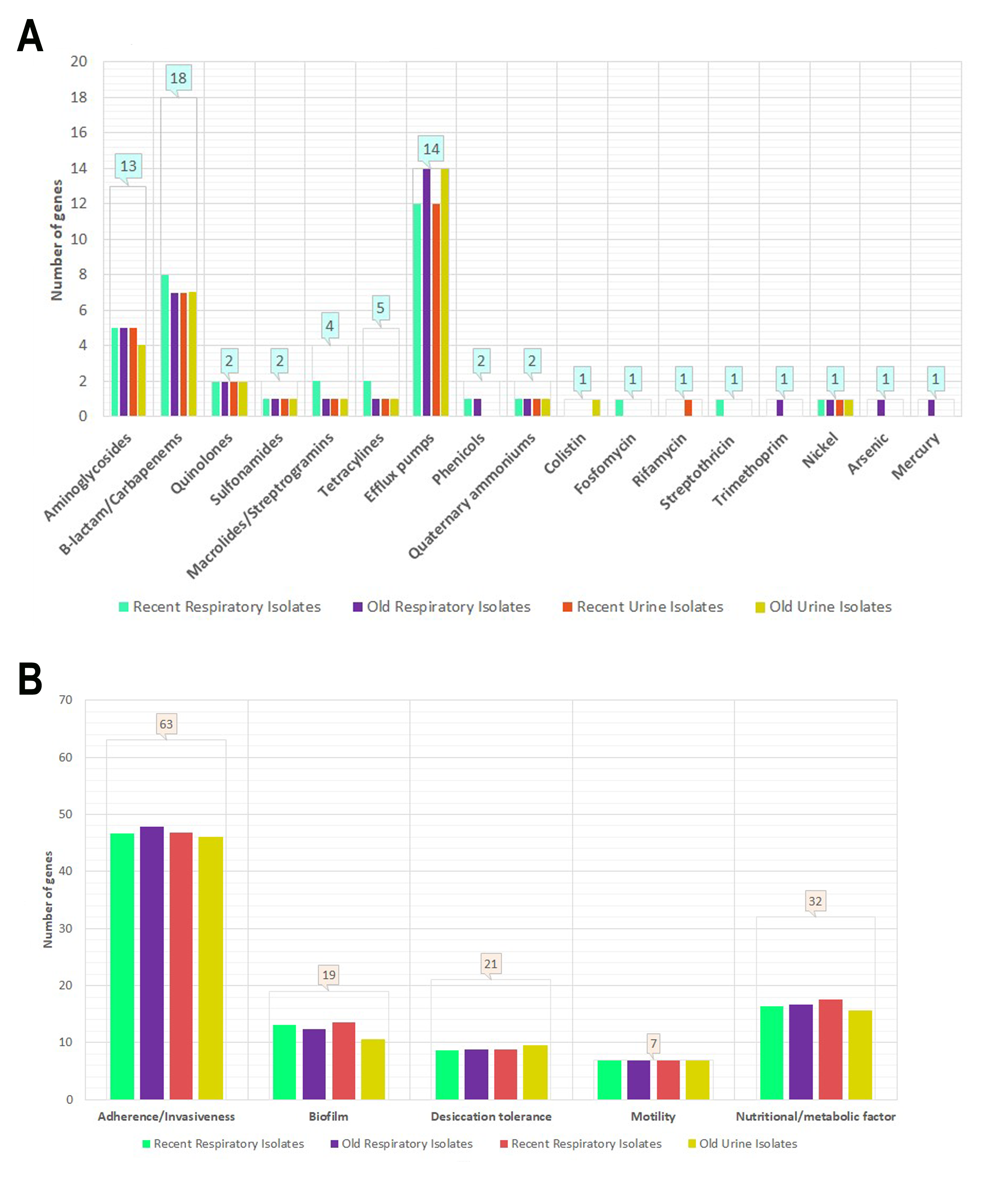

Supplement: Supplementary file 1 [file Image1.tiff]

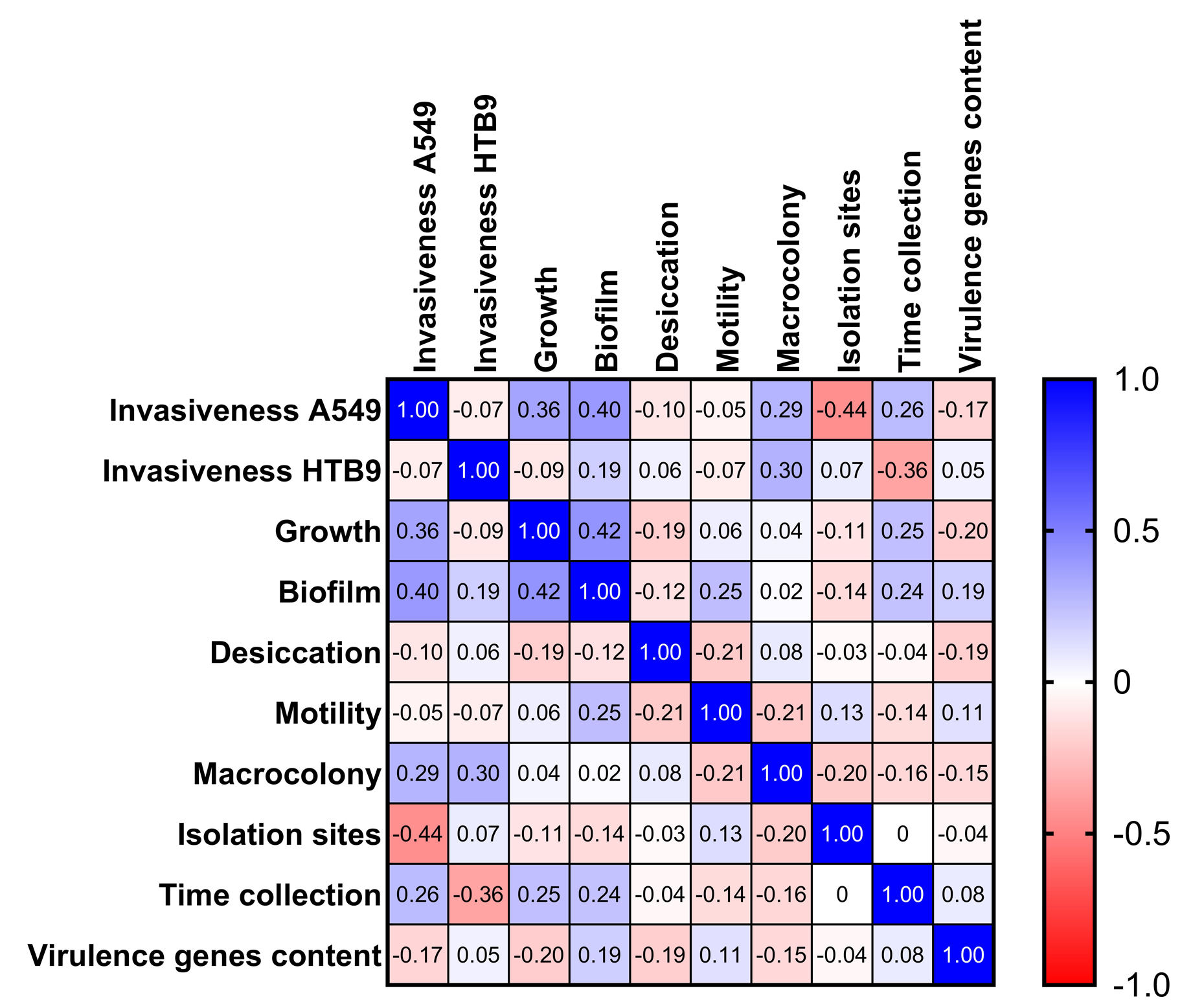

Supplement: Supplementary file 2 [file Image2.tiff]
