# Supplementary material for: A decade of genomic and phenotypic adaptation of carbapenem-resistant Acinetobacter baumannii
Source: Front Cell Infect Microbiol. 2025 Apr 30;15:1527488. doi: 10.3389/fcimb.2025.1527488 (PMC12075148; doi:10.3389/fcimb.2025.1527488)
Supplement: Supplementary file 3 [file Table1.docx]

Supplementary Material


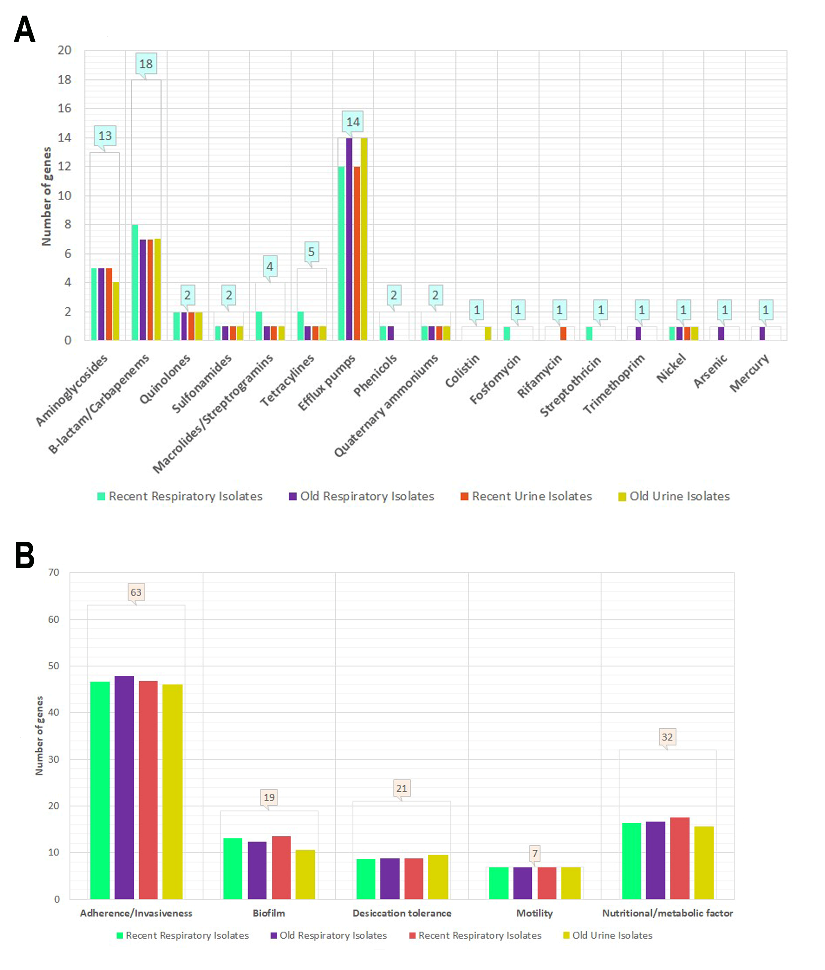


**Supplementary Figure S1**. **Comparison of antibiotic-resistance and virulence genes among isolates.** Each bar shows the average of the pooled number of genes encoding antibiotic resistance (A) and virulence (B), according to the type of isolate. Numbers at the top of the bar represents the total number of genes identified per functional category in all isolates. The graph was generated using Microsoft Excel**.**


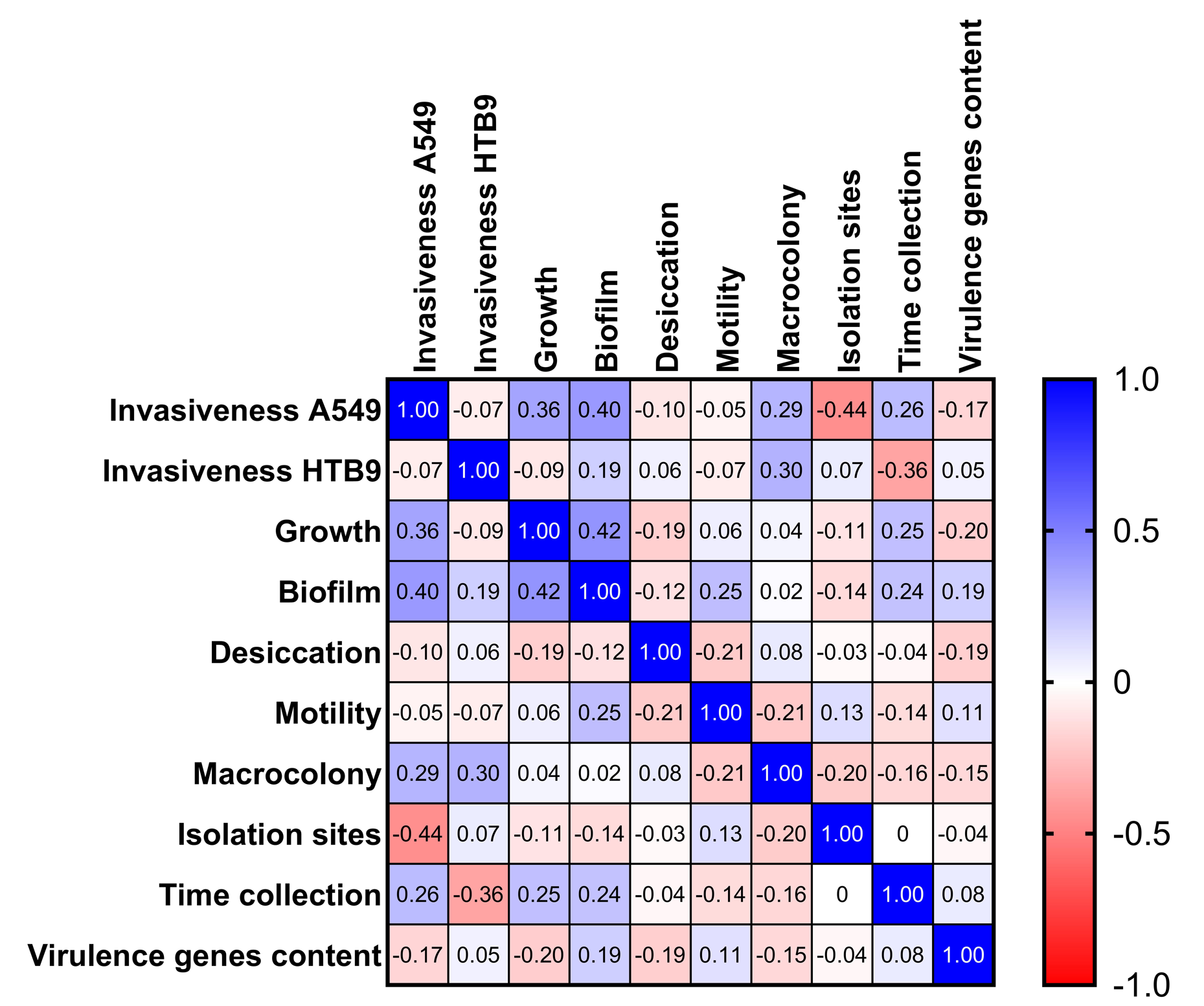


**Supplementary Figure S2. Phenotypic and genotypic correlation matrix of major phenotypes.** Parameters included in the analysis are reported. Data were transformed with the log(Y) function and analyzed using Pearson’s correlation. The colors represent Pearson’s correlation coefficient; its intensity depicts the coefficient’s value (shades of blue are positive correlations and shades of red are negative correlations). The graph was generated using GraphPad Prism version 10.3.1.
